# Genome-Wide In Silico Analysis of Microsatellite Loci in Rabbits

**DOI:** 10.3390/ani14243659

**Published:** 2024-12-18

**Authors:** Hosam M. Safaa, Mostafa Helal, Seif Yasser, Zahra Raafat, Habiba Ayman, Hasnaa Mostafa, Milena Bozhilova-Sakova, Dalia A. A. Elsayed

**Affiliations:** 1 Department of Biology, College of Science, University of Bisha, P.O. Box 551, Bisha 61922, Saudi Arabia; 2 Department of Animal Production, Faculty of Agriculture, Cairo University, Giza 12613, Egypt; 3 Biotechnology Program, Faculty of Agriculture, Cairo University, Giza 12613, Egypt; seifyasser13@gmail.com (S.Y.); zahraraafat638@gmail.com (Z.R.); biba.ayman14@gmail.com (H.A.); hasoo.mostafa@gmail.com (H.M.); 4 Agricultural Academy, Institute of Animal Science, 2232 Kostinbrod, Bulgaria; bojilova_milena@abv.bg; 5 Department of Poultry Breeding, Animal Production Research Institute, Agriculture Research Center, Dokki, Giza 12618, Egypt; daliasoud2000@gmail.com

**Keywords:** SSRs, genetic diversity, mining, rabbits, verification, population structure

## Abstract

This study aimed to characterize microsatellites in the rabbit genome using an in silico approach and to develop and validate microsatellite markers. Blood samples were collected from 15 Baladi rabbits and 18 New Zealand White (NZW rabbits). The GMATA software was used to define SSRs in the extracted sequences. Twelve primer pairs were used to validate the loci identified and the primers developed. The total number of the detected microsatellite loci overall chromosomes was 1,136,253. The di-nucleotide microsatellite repeats dominated and exceeded 88% of the detected microsatellites in all chromosomes. There were no microsatellites detected in mitochondrial DNA. The highest relative microsatellite abundance was obtained for chromosome 19, followed by 13 and 6. The highest estimated SSR density was obtained for chromosome 14, and the lowest was for mitochondrial DNA, followed by chromosome 13. The polymorphism was 81.63% and 75.51% for Baladi and NZW rabbits, respectively. The number of detected alleles ranged between two and seven alleles/loci, and polymorphic information content was from 35% to 71%. The AMOVA analysis showed that the total variance of all levels of population structure was 15.734. The results definitely confirmed higher genetic diversity in Baladi compared with NZW rabbits.

## Simple Summary

Rabbits have a very important value in meat production, especially within the Middle East and most of Asia. By employing sophisticated computer analysis, attempts have been made to identify specific genetic markers called microsatellites within the rabbit genome. Blood samples were collected from two breeds of rabbits: one was the local Baladi rabbit, and the other one was a commercial New Zealand White rabbit. It has identified over one million microsatellite markers, therefore proving that the Baladi rabbits were truly more genetically diverse than New Zealand Whites. With a breeding for higher genetic diversity, chances are that healthy rabbits can adapt more easily to changes in their living environment and are more resistant to infections. The results derived from such a study have implications in food security and sustainability through conservation of genetic variation, especially in native breeds. Therefore, the present work can be of great utility to breeders and farmers in the improvement of rabbit populations toward better productivity combined with animal welfare.

## 1. Introduction

Rabbits (*Oryctolagus cuniculus*) are significant contributors to meat production, particularly in the Middle East and south east Asia [1]. They are small animals that exist in over 305 breeds worldwide, and they largely differ genetically. Rabbits are one of the most recently domesticated animals [2], native to the Iberian Peninsula and southern France, and their distribution is now seen throughout the Mediterranean region and worldwide [3]. Rabbits have a relatively mid-sized genome, consisting of ~2.7 billion bp, with 20,612 protein-coding genes and 42,943 gene transcripts [4]. The rabbit genome comprises 21 autosomes and one sex chromosome. It contains a high level of GC bonding, having approximately 42% of the nucleotides as guanine and cytosine. Rabbits have a rather low genetic variation compared to other mammals, probably because of their history of domestication and selective breeding [5,6]. Significant synteny, or conservation of gene order, has been observed through comparative genomic studies between the rabbit genome and other mammals, especially rodents and primates [7,8].

Microsatellites, or simple sequence repeats (SSRs), are genetic markers that have been preferred by breeders and geneticists for decades [9]. They have been applied for various purposes including breed-specific and individual identification, construction of linkage maps, and positioning of quantitative trait loci [10,11]. Microsatellites are widely distributed in the coding to non-coding regions of the genomes of all eukaryotic species [11]. Though some repeat types show higher frequencies in certain genomic regions, the implications are far from being known, and studies on repeat density and distribution within the genome are foreseen to provide information on functional significance [12,13]. The complete genome sequences now publicly available for many organisms, including rabbits, have allowed genome-wide research as well as in silico approaches [3]. A wide range of microsatellite marker panels could be envisaged in studies on the genetics underlying diseases and traits, evolution, phylogenetic, and genomic-assisted breeding and selection in rabbit production [14].

Various methods could be implemented for the identification and isolation of microsatellites, including both in silico and laboratory-based approaches [15]. Standard laboratory techniques involve DNA extraction, amplification of microsatellite-specific PCR fragments, and separation of products on a gel or capillary electrophoresis [15]. This approach is relatively accurate but it is time-consuming, labor-intensive, and costly [16]. On the other hand, in silico approaches use bioinformatic tools for large-scale microsatellite detection [13]. Nevertheless, combining the two approaches leads to more reliable results.

Nowadays, in silico approaches have become highly favored for the detection of microsatellites, for many reasons. First, these methods became more sensitive and very accurate; they can detect even short and imperfect repeats that might be overlooked in traditional techniques [13]. The second reason is that the in silico tool is low-cost and faster compared to any other labor-intensive method in the laboratory without reagents and complicated workflows [13]. Third, they are capable of handling large-scale datasets; genome-wide microsatellite analyses become feasible [17].

For that reason, the current study aimed at marking and characterizing microsatellites in the entire rabbit genome using an in silico approach, and developing and validating designed microsatellite markers for the detected loci by exploring the genetic diversity and population structure of two rabbit breeds.

## 2. Materials and Methods

### 2.1. Ethical Approval

All the research procedures were approved by the Institutional Animal Care and Use Committee at Cairo University (IACUC-CU), approval number (CU/I/F/32/23).

### 2.2. Data Source

Reference sequences of twenty-two chromosomes, including twenty-one autosomes and the X chromosome of the rabbit (*Oryctolagus cuniculus*), in addition to the mitochondrial DNA sequence, were used to mine and search for microsatellites (SSRs). The reference genome was for Thorbecke inbred females, which is a rabbit breed that was completely sequenced and submitted in 2009 and is publicly available and accessed by the National Center for Biotechnology Information database (www.ncbi.nlm.nih.gov (accessed on 1 November 2023)), and the Ensemble genome database (www.ensembl.org (accessed on 1 November 2023)), both with assembly accession (GCA_000003625.1). We extracted the reference genome sequence of rabbits from the Ensemble database and used it in this study. The genome was assembled completely to 2737.46 Mb with 22 chromosomes and mitochondrial DNA, 3318 scaffolds, 2,737,490,501 bp, 20,612 coding genes, 8319 non-coding genes, 656 pseudogenes, and 51.853 gene transcripts.

### 2.3. In Silico Mining of Whole-Genome-Wide SSRs

The Genome-wide Microsatellite Analyzing Tool Package (GMATA, v21) was used to mine SSRs in the extracted sequences [17]. The choice of this program was based on some features such as providing SSR mining, statistical plotting, designing primers, polymorphism screening, and mapping. GMATA performs calculations faster for large genomes and provides more accurate results than many tools; the existing program can process large sequences of DNA, and is also simple to use, with a graphical user interface.

### 2.4. Validation

Twelve primer pairs (Table 1) were randomly chosen at different chromosomes to validate the loci identified and the primers developed. The sequences of the different primers are presented in Supplementary Table S1. Validation was carried out using polymerase chain reaction (PCR) amplification. Blood samples were collected from the ear vein of 33 rabbits of both sexes of two breeds, where 15 Baladi rabbits were sampled as a local breed and 18 New Zealand White (NZW) rabbits as a commercial breed. DNA isolation was performed using wizPrep Genomic DNA Kit (Wizbiosolutions Inc., Gyeonggi-do, Republic of Korea) according to the manufacturer’s protocol. The volume of the PCR reaction was 20 μL, consisting of 2 µL template DNA (~40–50 ng), 2 µL of primers (1 µL of each forward and reverse primer), 10 µL master mix, and 6 µL nuclease-free water. The PCR amplification cycle started with the initial denaturation step at 95 °C for 5 min, followed by 35 amplification cycles of denaturation at 95 °C for 32 s, annealing at 57–62 °C for 54 s, extension at 72 °C for 30 s, and final extension at 72 °C for 10 min. The PCR products were separated using electrophoresis on an 8% non-denaturing polyacrylamide gel. Genomic parameters including number of alleles, heterozygosity and number of effective alleles were calculated using GenAlex [18]. Multilocus genotypes (MLG), Shannon–Wiener index (H), Stoddart and Taylor’s index (G), Simpson’s index (λ), evenness index (E.5), and index of association (Ia) were calculated using diveRsity package of R [19]. F-statistics and principle coordinate analysis were calculated using GenAlex [18]. Polymorphic information content (PIC) was calculated for each marker using Cervus [20].

## 3. Results

### 3.1. Microsatellite Distribution

**Figure 1:**
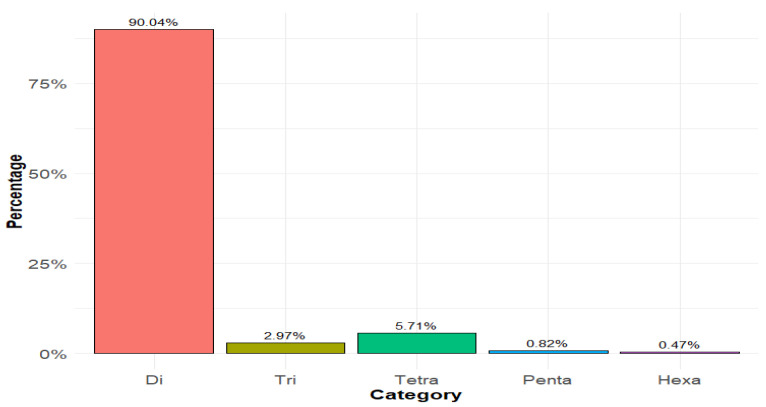
Distribution of the detected microsatellites overall in the chromosomes.

The numbers of microsatellite loci detected per chromosome are presented in Table 1. It can be inferred from the table that the GMATA software successfully detected microsatellite loci in the different chromosomes. The overall percentages of the detected microsatellites are presented in Figure 1: the di-nucleotide SSRs percentage was 90%, followed by tetra-nucleotide and tri-nucleotide, with percentages of 6% and 3%, respectively. The total number of the detected microsatellite loci overall in the chromosomes was 1,136,253.

**Figure 2:**
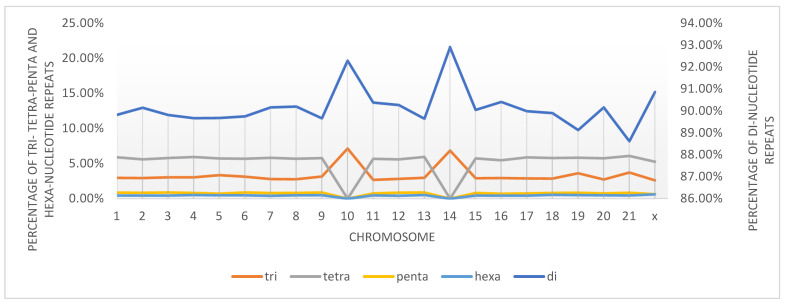
Percentages of K-mer nucleotide repeats detected for each chromosome of rabbit genome (Tri-tetra-penta and hexa-nucleotide repeats on Y axis, and dinucleotide repeat on Z axis).

The type and percentage of microsatellite repeats are also presented in Table 1, and the total percentage of each repeat is presented in Figure 2. The di-nucleotide microsatellite repeats dominated, and exceeded 88% of the detected microsatellites in all chromosomes. Tri-nucleotide repeats ranged between 2.6% and 7.2%. Although tetra-nucleotide repeats accounted for more than 5% of all chromosomes, they were not detected in chromosomes 10 and 14. All the other repeats were less frequent, and penta-nucleotide and hexa-nucleotide repeats were rare; the highest parentages were 89% in chromosome 3 and 0.62% in chromosome X, for penta-nucleotide and hexa-nucleotide, respectively. However, neither repeat was detected in chromosomes 10 and 14.

**Figure 3:**
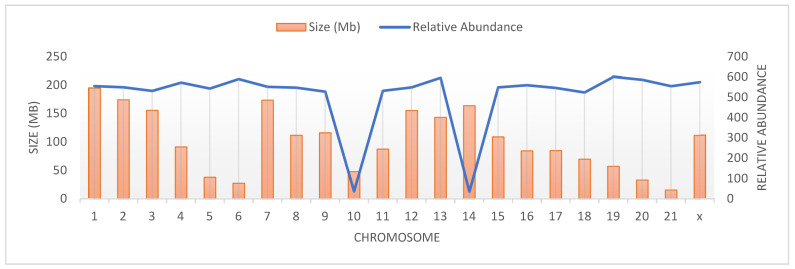
Size of the different chromosomes and relative abundance of the total detected microsatellites in the rabbit genome.

Table 2 shows the size (Mb) of different chromosomes. Chromosome 1 was the largest chromosome, followed by chromosomes 2, 7, and 14, while the smallest were chromosomes 21, 6, 20, and 5. Also, the reference sequence of mitochondrial DNA was too small (0.02 Mb), and, therefore, there were no microsatellites detected in mitochondrial DNA. The relative abundance of microsatellites is illustrated in Figure 3 and listed in Table 2, as well. Unexpectedly, the highest relative microsatellite abundance was not obtained for the largest chromosome (chromosome 1), but for chromosome 19, followed by chromosomes 13 and 6. Although the relative abundance is usually dependent on the size of the sequence analyzed, chromosome 1 was ranked as eighth in the row.

It should be noted that the number of SSRs showed a strong positive correlation with chromosome size. The correlation coefficient between chromosome size and the number of detected microsatellites was 0.814. The correlation between chromosome size and relative abundance was assessed as well, and was almost zero (−0.005). However, the correlation coefficient between chromosome size and repeat density was 0.367. The estimated density was low, and the lowest was zero, obtained for mitochondrial DNA, followed by chromosome 13 (0.00076), whereas the highest estimated SSR density was obtained for chromosome 14 (0.01372), followed by chromosome 10 (0.01291). It is well known that the study of repeat density and its distribution pattern helps in understanding their significance.

For the distribution of the detected microsatellite types, the values obtained for di-nucleotide repeat relative abundance were higher than other motifs on all chromosomes except for chromosomes 10 and 14. Similarly, the relative abundance of tetra-nucleotide repeats was higher than trinucleotide repeats for all chromosomes, except for chromosomes 10 and 14.

### 3.2. Design of the Primers

The number of primers designed for the detected markers is presented in Table 3. The average percentage of SSRs without primers was 0.03%. The percentages ranged between 77.01% on chromosome 14 and 99.7% on chromosome 6. The successful development of markers indicates the possibility of SSR application. All the primers are available at https://github.com/mosthamed/OryCun2.0_Rabbit_SSR (accessed on 24 October 2024).

### 3.3. Validation of Microsatellite Markers and Diversity Evaluation

**Figure 4:**
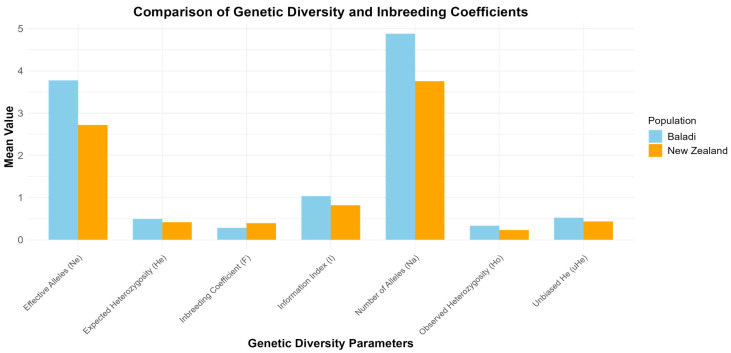
Comparison of genetic diversity parameters between Baladi and NZW populations.

All twelve markers were successfully amplified from the two studied breeds (PCR gel examples are presented in Supplementary Figure S1). The polymorphism was 81.63% and 75.51% for Baladi and NZW rabbits, respectively. The number of detected alleles ranged between 2 and 7 alleles per loci, and polymorphic information content (PIC) ranged from 35% to 71%, as shown in Table 1. Microsatellite analysis revealed differences in genetic diversity between the two populations of rabbits, Baladi and NZW (Figure 4). The details of genetic diversity parameters per locus are presented in Supplementary Table S2. The average number of alleles (Na) in the Baladi rabbits was 4.878, while the effective number of alleles (Ne) for the Baladi population was 3.775. Shannon’s information index (I) was 1.037. The observed heterozygosity (Ho) was 0.333, and the expected heterozygosity (He) was 0.498. The unbiased expected heterozygosity (uHe) was 0.522, while the coefficient of inbreeding was 0.285, hence indicating moderate inbreeding. In the NZW population, the number of alleles (Na) was 3.755, and the number of expected alleles (Ne) was 2.720. Shannon’s index was 0.822. The observed heterozygosity (Ho) in the NZW population was 0.231, while the expected (He) and unbiased heterozygosity (uHe) were calculated as 0.419 and 0.436, respectively. The inbreeding coefficient of the NZW rabbits was 0.395 which indicated higher inbreeding compared to the Baladi population.

Genotypic diversity analysis parameters are presented in Table 4. In the Baladi population, where all samples maintained distinct multi-locus genotypes, an eMLG of 15 was recorded. The Shannon–Wiener index (H) was 2.71, with a Stoddart and Taylor’s index (G) of 15 and a Simpson’s index (λ) of 0.933, indicating high genotypic diversity. The evenness index (E.5) was 1, and the expected heterozygosity (He) was 0.532. The index of association (Ia) was 1.14, and the standardized index of association (rD) was 0.0341.

In the NZW population, all 18 individuals had different MLGs, while the eMLG was 15. H = 2.89, G = 18, and λ = 0.944—an indication of high genotypic diversity. The evenness index (E.5) was 1, and He value was 0.436, while the index of association was higher at 1.7, correspond with the rD value of 0.0523, hence showing a slightly stronger linkage disequilibrium when compared to the Baladi population.

The overall population showed a total of 33 MLGs and an eMLG of 15. Also, the total Shannon–Wiener index was 3.5, G = 33, and λ = 0.970, showing very high overall genotypic diversity. The expected heterozygosity was 0.558, with an Ia of 1.48 and an rD of 0.0396.

As shown in Table 5, the average within-population inbreeding coefficient (F_IS_) was 0.338. The overall inbreeding coefficient (F_IT_) was 0.429. The fixation index (F_ST_) averaged at 0.172. Gene flow was estimated at 10.696. The analysis of molecular variance (AMOVA, Table 5) showed that 17% of the total genetic variation was contributed by variation among populations, with an estimated variance of 2.744. Variation among individuals within populations contributed to the highest part of 44% of the total variance, with an estimated variance of 6.869. Finally, 39% of the genetic variation came from within individuals, accounting for an estimated variance of 6.121. Aggregating these, the total variance was 15.734, which spanned all levels of population structure.

**Figure 5:**
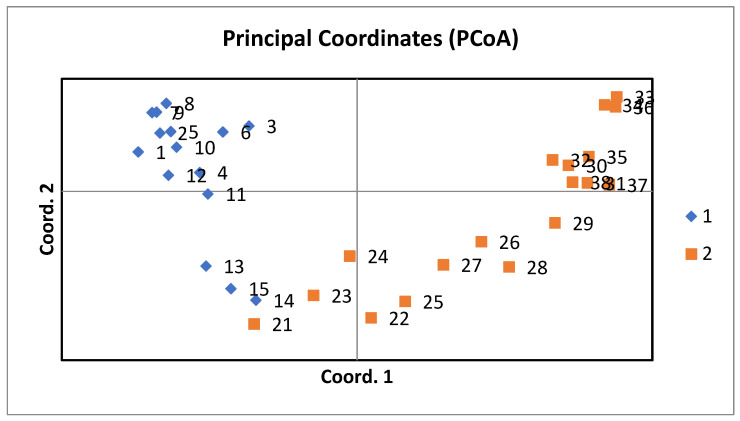
Principal coordinate analysis of the 2 population inferred by the designed microsatellite markers for New Zealand White (1) and Baladi (2) rabbits.

The Principal Coordinate Analysis (PCoA) results are presented in Figure 5. The first three axes outline a cumulative 52.59% of the variance. Axis 1 explains the highest percentage of variation, accounting for 29.88%. The second highest variation is explained by Axis 2 (14.93%). Therefore, those two axes cumulatively explain 44.81% of the total variance. Further, 7.78% is added by Axis 3, reaching a cumulative explained variance of 52.59%. In this PCoA plot, individuals from the two breeds tend to cluster together. Baladi rabbits fall within the top-left and lower-left quadrants, showing a close genetic affinity among the members of this breed. However, The NZW rabbits fall within the top-right and bottom-right quadrants, displaying their clear separation from those of the Baladi breed.

## 4. Discussion

Despite many studies being performed using in silico approaches and bioinformatic tools to characterize microsatellites in certain animal species such as duck and chickens [13,16,21,22], many animal species have not yet been subjected to such analyses. The current study analyzed the microsatellite characteristics of domestic rabbits for the first time. The obtained results indicate the effectiveness of GMATA in the detection of microsatellite loci on various chromosomes, with a total of 1,136,253 microsatellite loci being detected. This high number of detected loci points to the capability of GMATA in finding microsatellites efficiently, while such markers are basically indispensable in a wide range of studies from population genetics to genome mapping. The GMATA software was reported as a superior fast software for microsatellite mining [13,17,23].

A large proportion of the detected loci were dominated by dinucleotide SSRs. This pattern corresponds to those previously reported in the genome of various organisms, especially vertebrates, in which dinucleotide repeats were the most abundant, with the AC/GT repeats being at the top. The dinucleotide repeats were reported to be the most frequent motifs, and accounted for 71.7% of the detected microsatellites in catfish [24], and for 55.98 and 58.75% in Mallard and Muscovy ducks, respectively [13]. This high frequency could be justified by the fact that shorter repeat units were more susceptible to replication slippage, one of the main mechanisms of microsatellite mutation. For the majority of mammals, dinucleotide repeats were the dominant repeats, and were essentially represented by TG repeats [25]. In both pigs and men, TG-repeat microsatellites were 2.5–3 times more frequent than TC-repeat microsatellites. This difference was even greater in ruminants, with 5–10 times more TG repeats [26].

In contrast, trinucleotide and tetranucleotide repeats were much rarer in the identified loci. This was expected, since longer repeat motifs would be less common because of their relatively lower mutability and the possibility of stronger selective pressures against them, especially in coding regions. It might reflect specific characteristics of the chromosomes or structural limitations that hinder the formation of these repeats, or that were selectively eliminated during the process of evolution. Trinucleotide and tetranucleotide microsatellite repeats were distributed unevenly across vertebrate genomes, and show over-representation in the coding regions [27]. Concerning this, it was reported that selection can shift the distribution of trinucleotide repeats in different organisms [28].

The data also showed that chromosome size did not correlate with the number of identified microsatellites, something that was quite astonishing and again demonstrated the intricacy of the distribution of microsatellites in rabbits. This contradicted the assumption that larger chromosomes would automatically have more microsatellites. The correlation between chromosome size and detected microsatellites was reported earlier in different species including ducks [13], crassostrea [29], and cattle [30]. However, the current study indicated a difference in chromosomal architecture, with smaller chromosomes in rabbits now having more non-coding repetitive sections where microsatellites can proliferate without deleterious effects. Again, this could indicate increasing recombination rates or more open chromatin structures, which would facilitate the expansion of the microsatellite repeats [31].

The results in the current study also agreed with the knowledge on mtDNA structure and function, in that mtDNA does not harbor microsatellites. In rabbits, mtDNA is small in size, highly conserved, and mainly encodes genes central to oxidative phosphorylation [32]. Thus, mtDNA is under strong purifying selection, together with a higher mutation rate, particularly within microsatellite regions. This conservation may prevent the formation or persistence of microsatellites [33]. The absence of microsatellites in mtDNA thus further supports the view of mtDNA being a streamlined highly functional genetic element, as compared with the nuclear genome, which is more dynamic and mutable [34].

These findings added to the growing awareness of the complexity underlying the distribution of microsatellites across chromosomes. While chromosome size is one factor, it clearly shows that other genomic attributes such as gene density and recombination hotspots are also as important. Further studies are needed to establish functionality for microsatellite-rich regions of smaller chromosomes, where the relative microsatellite densities are greater. The absence of microsatellites in mtDNA and the uneven distribution of various repeat types in chromosomes open up even more interesting pathways for further studies, mainly concerning the evolution of microsatellites in different genomic contexts.

Such in silico identification is effective but often gives false positives, especially in regions containing repeats or low complexity. Hence, it is important that these microsatellite loci are experimentally validated for their biological significance. Also, such a huge number of loci need consideration in their practical applications, especially in genetic studies, to balance both quantity and quality of marker selection.

Despite the huge number of markers designed successfully, a number of loci failed to design any primer. This limitation could be related to particular problems at the target locus, unsuitable flanking regions, or even sequences which were not appropriate for optimum primer design criteria, including inappropriate GC content or melting temperatures. For this, a validation step was performed, as all the twelve microsatellite markers yielded successful amplifications, which indicated the success of the primer design process.

Genetic diversity and population structure were previously described for the Baladi and NZW rabbits, based on microsatellite analysis [35]. Observed genetic diversity between the Baladi and NZW populations agreed with expectations, since local breeds, such as the Baladi rabbit breed, usually possess higher genetic diversity compared to an intensively bred commercial line like NZW rabbits [36]. This is generally attributed to the more uncontrolled breeding in local breeds, allowing for a greater number of alleles and wider genetic variation than that experienced in selective breeding and possible genetic bottlenecks in commercial lines [9].

These findings were further supported by the higher levels of polymorphism recorded in Baladi rabbits, at 81.63% against the NZW rabbits, which recorded 75.51%. Local breeds, such as Baladi, are better adapted to variable and diverse environmental conditions, and hence are expected to maintain higher levels of genetic diversity, an important determinant of fitness, and, lately, also a factor in resilience against environmental changes [35]. Commercial breeds, like NZW, on the other hand, have usually undergone selective breeding, entailing a reduction in genetic variation due to either genetic drift, inbreeding, or founder effects [37]. The inbreeding coefficient was relatively higher in the NZW population, giving further support for the reduced genetic variability reported in other commercial livestock species as well, and likely a consequence of the intensive selection and closed breeding programs.

The observed heterozygosity was significantly lower in the NZW population compared to the heterozygosity observed for the Baladi rabbits because the latter has lost so much genetic variability through artificial selective breeding [38]. Observed, expected, and unbiased heterozygosity values were estimated to be 0.681, 0.842, and 0.872 in Soviet Chinchilla and 0.665, 0.849, and 0.880 in Californian White rabbits, respectively. [39]. The current estimates of expected heterozygosity in both populations, however, gave the indication that genetic diversity, at least in the Baladi breed, was maintained, though there was still some discrepancy between observed and expected heterozygosity within the two populations, as previously described [35]. Especially for the NZW rabbits, this discrepancy may be due to the Wahlund effect [40], where different allele frequencies across subpopulations decrease the observed heterozygosity, compared with the expected ratio based on the Hardy–Weinberg equilibrium [41].

Results from the genotypic diversity analysis further strengthen the results obtained for the genetic diversity and polymorphism. These results thus indicated that both populations had a high genotypic diversity; however, the low index of association in the Baladi rabbits suggested linkage disequilibrium to be much weaker as compared to the NZW population [42]. This relatively low disequilibrium could mean that the mating in this breed was more random, with less selection pressure, while the NZW breed might have been under stronger selective pressure, which accounted for the higher observed linkage disequilibrium. These results agreed with reports from other studies where the extent of LD is relatively low in local breeds, and correspondingly associated with a higher genetic diversity and less intense selection [43].

Results from the AMOVA showed that the majority of genetic variation was partitioned within individuals and among individuals within populations, leaving only a small proportion of 17% to be distributed between populations. This distribution of variance was in agreement with expectations for domestic animal populations, where substantial diversity within a population was common due to the complex breeding systems and the genetic histories of different breeds. These results were supported by those previously obtained for domestic rabbit breeds [44]. The fixation index consequently points toward a moderate genetic differentiation between the two populations. A lower F_ST_ value of 0.04 was obtained previously for NZW and APAU Black rabbits [45].

Further confirmation came from the genetic differentiation highlighted by the results of principal coordinates analysis. The clear grouping of Baladi and NZW rabbits supports the fact that these populations have pursued an independent evolutionary path, driven by the probable reason of their breeding objectives, Baladi rabbits being influenced by natural and local selection, while NZW rabbits have been under artificial selection for growth rate, meat production, and docility.

The overall results showed that the Baladi population possessed greater genetic diversity, with lower inbreeding compared to NZW, as reported previously [46]. This pointed to the fact that breeds subjected less to intensive selection retain more genetic variability, which can be seen in local breeds, and this might result in a definite advantage for long-term adaptability and resilience [47].

In this regard, the genetic data obtained point to the fact that NZW is an inbred population with reduced genetic diversity. Such reduced genetic variability increases the risk of inbreeding depression, which can manifest as compromised fertility, diminished disease resistance, and overall reduced fitness. These vulnerabilities underline the need for strategies to mitigate these effects in intensively bred animals.

From the perspective of breeding management, this might well call for strategies aimed at maintaining genetic diversity through outcrossing or controlled mating, which could reduce inbreeding and preserve the long-term viability of commercial rabbit breeds such as NZW. These results therefore underscored the need to preserve genetic diversity in domestic breeds, both for improving adaptability to changing climate conditions and maintaining good health and high productivity within the population. The slight genetic differentiation between Baladi and NZW rabbits essentially reflects different breeding management, and this should form the basis for future strategies in breeding and conservation, to balance selection with genetic resource preservation. Additionally, as the validated microsatellite loci exhibited high polymorphism, they can be used in a wide range of genetic applications. These include parentage testing and pedigree reconstruction, especially in managed breeding programs. Such applications may enable the accurate tracking of the relationships of parents, genetic diversity assessment, and verification of breeding records. The markers have the potential for breed discrimination and, as such, would be highly useful in cloned-animal production verification. Future studies should investigate the parentage assignment power of these loci and determine their exclusion probabilities across diverse populations. This would allow the establishment of a robust minimal set of markers for pedigree testing.

## 5. Conclusions

This study achieved its objectives by identifying and validating microsatellite loci in the rabbit genome, providing a robust tool for assessing genetic diversity and population structure. The results showed that the Baladi rabbits had higher genetic diversity and lower inbreeding compared to the NZW rabbits, which presented reduced variability and higher inbreeding coefficients. These findings not only underscore genetic differences between the two populations, but also validate the utility of markers for applications like genetic diversity monitoring, population management, and, potentially, pedigree verification. Whereas the present study has focused on the characterization of markers, future research could use these results to explore breeding strategies and minimize inbreeding depression in an intensively bred population like NZW rabbits.

## Figures and Tables

**Table 1:** Number and percentage of the microsatellite loci detected for each chromosome of rabbit genome.

chr #	Motif (-mer)				
	Di	Tri	Tetra	Penta	Hexa
1	97,234 (89.82)	3210 (2.96)	6386 (5.89)	936 (0.86)	484 (0.44)
2	86,368 (90.14)	2824 (2.94)	5375 (5.61)	815 (0.85)	423 (0.44)
3	74,336 (89.81)	2516 (3.04)	4801 (5.80)	737 (0.89)	372 (0.44)
4	46,899 (89.67)	1590 (3.04)	3109 (5.94)	427 (0.81)	271 (0.51)
5	18,515 (89.70)	694 (3.36)	1184 (5.73)	147 (0.71)	101 (0.48)
6	14,562 (89.78)	509 (3.13)	924 (5.69)	145 (0.89)	78 (0.48)
7	86,449 (90.17)	2681 (2.79)	5583 (5.82)	770 (0.80)	388 (0.40)
8	55,261 (90.21)	1703 (2.78)	3491 (5.69)	504 (0.82)	297 (0.48)
9	55,042 (89.67)	1940 (3.16)	3550 (5.78)	542 (0.88)	305 (0.49)
10	1665 (92.80)	129 (7.19)	0	0	0
11	42,132 (90.40)	1250 (2.68)	2647 (5.67)	354 (0.75)	221 (0.47)
12	76,989 (90.28)	2414 (2.83)	4783 (5.60)	734 (0.86)	354 (0.41)
13	76,630 (89.66)	2545 (2.97)	5088 (5.95)	755 (0.88)	449 (0.52)
14	5379 (93.12)	397 (6.87)	0	0	0
15	53,929 (90.07)	1743 (2.91)	3444 (5.75)	492 (0.82)	266 (0.44)
16	42,780 (90.44)	1389 (2.93)	2588 (5.47)	336 (0.71)	207 (0.43)
17	41,801 (90.02)	1345 (2.89)	2735 (5.89)	351 (0.75)	201 (0.43)
18	32,895 (89.94)	1049 (2.86)	2123 (5.80)	301 (0.82)	203 (0.55)
19	30,693 (89.17)	1250 (3.63)	2010 (5.83)	291 (0.84)	174 (0.50)
20	17,560 (90.25)	533 (2.73)	1121 (5.76)	148 (0.76)	94 (0.48)
21	7678 (88.83)	323 (3.73)	528 (6.10)	74 (0.85)	40 (0.46)
X	58,301 (90.86)	1676 (2.61)	3380 (5.26)	409 (0.63)	399 (0.62)
MT	0	0	0	0	0

**Table 2:** Chromosome size, the total number of base pairs, relative abundance, and estimated repeat density of the detected microsatellite loci in the rabbit genome.

chr #	Size (Mb)	Total SSR	Relative Abundance	Number of Base Pairs	Estimated Repeat Density
1	194.85	108,250	555.56	237,226	0.00082
2	174.33	95,805	549.56	209,321	0.00083
3	155.69	82,762	531.58	181,341	0.00086
4	91.39	52,296	572.23	114,765	0.00080
5	37.99	20,641	543.33	45,189	0.00084
6	27.50	16,218	589.75	35,540	0.00077
7	173.68	95,871	552.00	209,451	0.00083
8	111.80	61,256	547.91	133,897	0.00083
9	116.25	61,379	527.99	134,644	0.00086
10	48.00	1794	37.38	3717	0.01291
11	87.55	46,604	532.31	101,698	0.00086
12	155.35	85,274	548.92	186,146	0.00083
13	143.36	85,467	596.17	187,716	0.00076
14	163.90	5776	35.24	11,949	0.01372
15	109.05	59,874	549.05	130,919	0.00083
16	84.48	47,300	559.90	103,001	0.00082
17	85.01	46,433	546.21	101,538	0.00084
18	69.80	36,571	523.94	80,152	0.00087
19	57.28	34,418	600.87	75,675	0.00076
20	33.19	19,456	586.20	42,507	0.00078
21	15.58	8643	554.75	19,047	0.00082
X	111.70	64,165	574.44	139,589	0.00080
MT	0.02	0	0	0	0.00000

**Table 3:** Number of primers designed for SSR amplification.

chr #	Total SSR	Total SSR with Primers	Percentage
1	108,250	107,538	99.34%
2	95,805	95,130	99.30%
3	82,762	82,210	99.33%
4	52,296	51,954	99.35%
5	20,641	20,514	99.38%
6	16,218	16,162	99.65%
7	95,871	95,144	99.24%
8	61,256	60,753	99.18%
9	61,379	60,992	99.37%
10	1794	1470	81.94%
11	46,604	46,193	99.12%
12	85,274	84,535	99.13%
13	85,467	84,969	99.42%
14	5776	4448	77.01%
15	59,874	59,316	99.07%
16	47,300	47,014	99.40%
17	46,433	46,090	99.26%
18	36,571	36,361	99.43%
19	34,418	34,258	99.54%
20	19,456	19,355	99.48%
21	8643	8586	99.34%
X	64,165	63,779	99.40%
MT	0	0	0

**Table 4:** Genotypic diversity analysis parameters.

Breed	Poly%	MLG	eMLG	SE	H	G	Lambda	E.5	Ia	rbarD
Baladi	81.63	15	15	0.00 × 10^0^	2.71	15	0.933	1	1.14	0.0341
NZW	75.51	18	15	1.05 × 10^−7^	2.89	18	0.944	1	1.7	0.0523
Total	--	33	15	0.00 × 10^0^	3.5	18	0.970	1	1.48	0.0396

Poly%: Percentage of polymorphic loci; MLG: Number of multilocus genotypes; eMLG: Expected multilocus genotypes; SE: Standard error; H: Shannon diversity index; G: Genotypic diversity index; Lambda (λ): Simpson’s index of genotypic diversity; E.5: Genotypic evenness index; Ia: Index of association; rbarD: Standardized index of association.

**Table 5:** AMOVA analysis and F-statistics of the studied breeds.

AMOVA Analysis			F-Statistics		
Source	Estimated Variance	%		Mean	SE
Among Breeds	2.744	17%	F_IS_	0.338	0.087
Among Individuals	6.869	44%	F_IS_	0.429	0.078
Within Individuals	6.121	39%	F_ST_	0.172	0.028
Total	15.734	100%	N_m_	10.696	5.985

## Data Availability

All data are included in the manuscript and Supplementary Materials.

## Supplementary Materials

The following supporting information can be downloaded at: https://www.mdpi.com/article/10.3390/ani14243659/s1, Table S1: Sequences and characteristics for the selected microsatellite primers; Table S2: Genetic diversity parameters, per locus, of the two rabbit breeds; Figure S1: Examples of PCR amplifications of locus 1 in Baladi (a) and New Zealand White rabbit (b) rabbits.
