# Supplementary material for: Genome-Wide In Silico Analysis of Microsatellite Loci in Rabbits
Source: Animals (Basel). 2024 Dec 18;14(24):3659. doi: 10.3390/ani14243659 (PMC11672705; doi:10.3390/ani14243659)
Supplement: Supplementary file 1 [file animals-14-03659-s001.zip › animals-3348698-supplementary.pdf]

Supplementary Table (S1). Sequences and characteristics for the selected microsatellite primers

Chr #	Primer sequence (F)	Primer sequence (R)	Tm	Allele range	Number of alleles	PIC
1	TTCTAGGCCAAAAGCTGGAA	CTGTCCACTCTGCCTGTCAA	59	135-284	7	58
2	GAGGTGAACCAATGGAAGGA	CCTGGACTGGTGGAGTTTTC	57	220-250	5	44
3	CGAACCTCAATAACCCGAGA	GCCTGCTCTTTGTCTTGCTC	60	151-169	4	62
4	GTGCTTCCAGTGAGGGTGAT	CCAGAAGCTGGGTCTCTGTC	61	152-181	5	66
7	ACCACCTCTGAGTGAACGA	TAAAGGCAGAGATGCCAGGT	58	133-300	5	54
8	GGCCGCATGATGAGAGATTA	TTTGGAGTGTGAACCAGCAG	62	117-519	4	51
9	GGAAGAAACTCCTGGCTCCT	TAGCCCCTACACACCACACA	59	50-494	3	57
11	CGCTGGCCAATAATCAACTC	ATCTCGCTGCTAATGCACCT	62	68-129	4	44
12	GTTCAGCCTGCAAAAGGAAG	AGTGAGCCAGTGGATGGAAG	59	130-207	2	42
13	ACATGGGAAACCTGGATTGA	TAGCGCTCTGTGAATGTTGC	61	218-588	5	71
15	GCTAGCTCCAGCAGACTTCA	GCTTCATATCCCCTGCTTCA	58	51-198	4	58
16	CCTGAGGGTGTGTGAGTGTG	GCTGCCGTTCACCCTATGTA	60	206-494	2	35

Supplementary Table S2: Genetic diversity parameters, per locus, the two rabbit breeds

Locus	Na	Ne	I	Ho	He	uHe	F
			**Baladi Rabbits**				
**Locus 1**	6.600	5.772	0.567	0.435	0.378	0.392	-0.124
**Locus 2**	4.600	3.529	0.397	0.264	0.281	0.291	0.068
**Locus 3**	3.750	3.689	0.503	0.373	0.358	0.371	-0.047
**Locus 4**	4.600	3.513	0.389	0.413	0.274	0.284	-0.449
**Locus 5**	5.000	4.516	1.133	0.238	0.561	0.592	0.576
**Locus 6**	3.700	2.296	1.437	0.342	0.675	0.703	0.507
**Locus 7**	2.667	2.269	1.242	0.167	0.537	0.559	0.661
**Locus 8**	3.500	3.848	1.792	0.571	0.660	0.691	0.157
**Locus 9**	2.000	1.735	1.205	0.063	0.554	0.587	0.925
**Locus 10**	2.750	2.045	0.671	0.250	0.371	0.394	0.333
**Locus 11**	3.750	2.517	2.481	0.423	0.903	0.938	0.534
**Locus 12**	2.000	1.952	1.785	0.115	0.787	0.872	0.873
			**New Zealand Rabbits**				
**Locus 1**	4.857	3.766	0.523	0.389	0.353	0.363	-0.058
**Locus 2**	3.600	2.384	0.327	0.235	0.220	0.227	0.014
**Locus 3**	1.750	1.736	0.516	0.347	0.372	0.382	0.067
**Locus 4**	3.600	2.477	0.367	0.400	0.256	0.263	-0.511
**Locus 5**	4.000	2.171	0.894	0.011	0.459	0.477	0.896
**Locus 6**	3.250	2.279	0.347	0.000	0.179	0.184	1.000
**Locus 7**	2.333	1.664	0.580	0.356	0.302	0.312	0.069
**Locus 8**	3.750	3.099	1.295	0.188	0.609	0.634	0.579
**Locus 9**	2.000	1.357	1.115	0.083	0.435	0.448	0.809
**Locus 10**	4.250	2.396	0.955	0.116	0.470	0.489	0.730
**Locus 11**	3.500	2.723	2.263	0.293	0.880	0.921	0.670
**Locus 12**	1.600	1.571	1.589	0.167	0.778	0.868	0.793

Na: Number of Alleles; Ne: Effective Number of Alleles; I: Shannon-Wiener Diversity Index; Ho: Observed Heterozygosity; He: Expected Heterozygosity; uHe: Unbiased Expected Heterozygosity; F: Inbreeding Coefficient

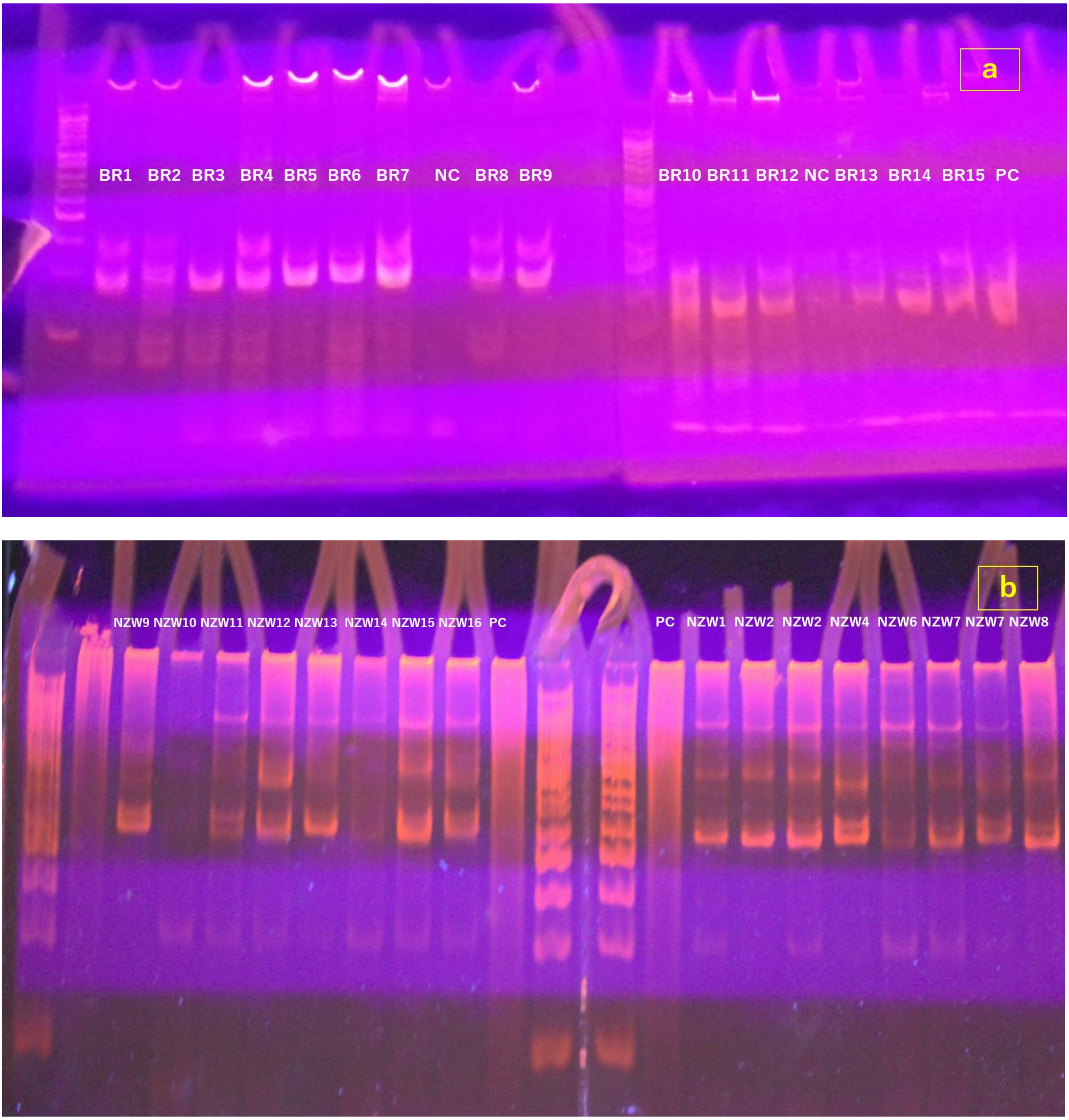

Supplementary Figure S3: Examples of PCR amplifications of locus 1 in Baladi (a) and New Zealand White rabbit (b) rabbits PC is the positive control and NC is the negative control
